# Congenital Cyst of Stomach

**Published:** 1916-03

**Authors:** 


**Congenital Cyst of Stomach.** Wm. Lyon, Jackson, Jour. Mich. State Med. Soc., Jan. The female infant seemed normal at birth and weighed 7 pounds. Two days later vomiting began and the child nursed poorly. 19 days after birth, the weight had diminished to 5¾ pounds, and the general condition was bad. A mass larger than a goose egg was found in the upper abdomen, freely movable. It was tense and fluctuating. Lavage of the stomach brought away brownish mucus but did not affect the tumor. Puncture brought away 4 ounces of clear glairy fluid, the last being quite thick. The empty sac was still palpable. Improvement occurred but the fluid reaccumulated and was twice removed by tapping, with the same results. One month after birth, operation was done. The cyst was attached along the greater curvature, extending slightly beyond the pylorus upon the duodenum. The wall was thick and friable, and the pylorus was cut into and was with difficulty closed again, during the operation. Death followed. The histologic structure of the cyst resembled that of the gastric wall, but with thinner and smoother mucosa. When full, the cyst caused pyloric obstruction.

